# Mental health needs in the acute and subacute phases of the 2024 Noto Peninsula earthquake: Emergency Medical Information System data analysis in Disaster Psychiatric Assistance Team activities

**DOI:** 10.1002/pcn5.70085

**Published:** 2025-03-17

**Authors:** Yasuto Kunii, Yumiko Hamaie, Mizuki Hino, Yusuke Utsumi, Yasuhisa Fukuo, Hiroaki Tomita

**Affiliations:** ^1^ Department of Disaster Psychiatry, International Research Institute of Disaster Science Tohoku University Miyagi Japan; ^2^ Department of Psychiatry Tohoku University Hospital Miyagi Japan; ^3^ Disaster Psychiatric Assistance Teams (DPATs) Secretariat, Commissioned by the Ministry of Health, Labor and Welfare Tokyo Japan; ^4^ Psychiatory Department Fujita Health University Hospital Aichi Japan; ^5^ Shinkeikahamamatsu Hospital Shizuoka Japan; ^6^ Department of Psychiatry Graduate School of Medicine, Tohoku University Miyagi Japan

**Keywords:** Disaster Psychiatric Assistance Team, earthquake, Emergency Medical Information System, mental health, resilience

## Abstract

**Aim:**

As it is difficult to examine the mental health of disaster victims immediately after a disaster through the rigorous procedures required for academic papers, the overall mental state of victims immediately after a disaster is not well understood. Therefore, this study aimed to investigate the actual mental health needs of victims during the transition from the acute and subacute phases to the chronic phase of the 2024 Noto Peninsula earthquake (NPE).

**Methods:**

We obtained Emergency Medical Information System (EMIS) data for a period of ∼1 month during Disaster Psychiatric Assistance Team support from outside the prefecture in Suzu City and Noto Town. We then extracted the following variables of the supportees from the EMIS data: age, consultation stage, content of response, psychiatric treatment history, and diagnostic classification. We compared the variables between the 2 weeks immediately after the disaster (Phase 1) and the following 2 weeks (Phase 2).

**Results:**

New consultations started mostly in Phase 1 and dropped in Phase 2. Consultations were initially dominated by victims with existing psychiatric disorders, but those on dementia‐related problems and direct stress reactions to the disaster increased over time.

**Conclusion:**

Information accumulated in the EMIS was useful for providing an overview of the mental state of communities affected by the 2024 NPE. The extracted findings may be useful for planning mental health measures for affected communities and preparing for future disasters, which may improve community resilience in affected areas.

## Graphical abstract

We investigated the actual mental health needs of victims during the transition from the acute and subacute phases to the chronic phase of the 2024 Noto Peninsula earthquake by using Emergency Medical Information System data for a period of ∼1 month during Disaster Psychiatric Assistance Team support. We compared the variables between the 2 weeks immediately after the disaster (Phase 1) and the following 2 weeks (Phase 2) and found that consultations were initially dominated by victims with existing psychiatric disorders, but those on dementia‐related problems and direct stress reactions to the disaster increased over time. These findings may be useful for planning mental health measures for affected communities and preparing for future disasters, which may improve community resilience in affected areas.

## INTRODUCTION

Japan is one of the world's most earthquake‐prone countries. In recent years, large‐scale natural disasters, such as typhoons and floods caused by linear precipitation zones, have occurred every year, depending on the location, topography, geology, and weather.^1^ Although records of major earthquakes and tsunamis have existed in Japan for a considerable time, it was not until the 1995 Great Hanshin‐Awaji Earthquake (GHAE) that the importance of mental health during and after disasters drew attention from mental health workers and society.^2^ Following the subsequent 2007 Chūetsu Offshore Earthquake,^3^ the consensus on the importance of mental health care for communities affected by a disaster was shared around the country, and several teams took part in mental health support activities in the area devastated by the 2011 Great East Japan Earthquake (GEJE).^4^ However, there was no systematic support system to organize these postdisaster mental health care activities and related information coordinated with the total disaster response system at the time, which resulted in problems such as delays in rescue for some psychiatric hospitals and an imbalance in support among affected regions. Reflecting on the problems that arose during relief efforts after the 2011 GEJE, the Disaster Psychiatric Assistance Team (DPAT) was organized in 2013.

The DPAT, consisting of psychiatrists, nurses, and logistics, supports disaster‐affected psychiatric hospitals in transporting patients to safe places or caring for remaining patients, assessing local psychiatric needs, and providing psychiatric medicine and mental health support in cooperation with disaster medical assistance teams, other support teams, and local psychiatric facilities, thereby fulfilling the function of responding to the rapidly increasing mental health needs in disaster‐affected areas.^5^, ^6^ With the organization of the DPAT, a system was established to clarify the chain of command and order immediately after a disaster, consolidate information, build a system of coordination within the overall framework of disaster medical support, and provide frameworks for pre‐registration and training. Subsequently, the DPAT was dispatched to areas affected by earthquakes, volcanic eruptions, typhoons, heavy rains, landslides, avalanches, and the COVID‐19 pandemic. As an example of the improved aspects of disaster response achieved by the DPAT, the transportation of patients from damaged psychiatric hospitals to safe areas was well organized in the 2016 Kumamoto earthquake compared with those in the 2011 GEJE.

Earthquakes have been occurring intermittently in the Noto Peninsula of the Ishikawa Prefecture in Japan since approximately 2018, and seismic activity has been particularly active since December 2020. Amidst these conditions, an earthquake of magnitude (M) 5.5 centered on the Noto Peninsula occurred at 16:06 h on January 1, 2024. Four minutes later, at 16:10 h on the same day, an M7.6 earthquake occurred, with a maximum Japan Meteorological Agency Seismic Intensity of 7. The M7.6 earthquake was the largest earthquake observed on the Noto Peninsula since 1885, when records started to be kept, and was larger than the M7.3 in the 1995 GHAE and the 2016 Kumamoto earthquake. Damage caused by the tsunami was confirmed in three municipalities, namely Suzu City, Noto Town, and Shiga Town. The flood depth in Suzu City, which had a particularly large flooded area, was estimated to have reached ∼4 m. As of April 16, 2024, 245 deaths were confirmed and 323 people were seriously injured because of the earthquake. In the affected area, 8536 houses were completely destroyed and 19,015 were partially destroyed. Water was cut off in ∼5310 houses, and 5449 people remained displaced in 319 evacuation centers.^7^

**Figure 1:**
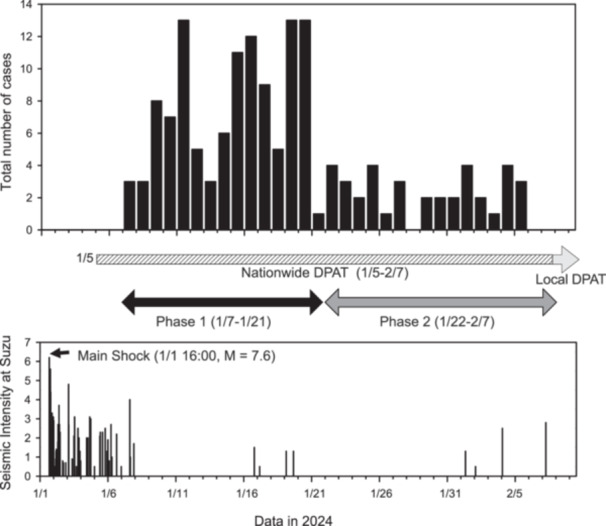
Number of cases of mental health support conducted by the Disaster Psychiatric Assistance Team (DPAT) displayed in a time series following the earthquake in Suzu. DPAT activity period, durations of Phase 1 and Phase 2, and seismic intensity data in Suzu are also included.

After the earthquake, following a request from the DPAT secretariat of the Ministry of Health, Labour and Welfare (MHLW) and Ishikawa Prefecture to dispatch a DPAT advance team, a total of 116 advanced DPATs from 43 prefectures, including Ishikawa Prefecture, were dispatched to the disaster‐affected area from all over Japan by February 7, 2024, when DPAT activities started to be managed merely by the local teams in Ishikawa Prefecture, and support from other prefectures was no longer needed.^8^ The authors participated in the third Miyagi Prefecture DPAT dispatch (February 1, 2024–February 7, 2024) as part of a mixed team from Tohoku University and the Miyagi Psychiatric Center. The team was dispatched when the disaster‐affected areas were moving out of the acute phase into the subacute and chronic phases (Figure 1), various stress reactions due to the burden of inconvenient evacuation were observed, and the problem of fatigue among supporters became apparent.^6^, ^9^, ^10^, ^11^

It is obvious that during a natural disaster that causes tremendous damage, affected people are placed in an environment that is clearly different from normal times, a situation that raises concerns about the impact on the medical supply and mental health of the victims. Prevalence of psychological distress, depressive symptoms, and post‐traumatic stress reactions has been widely observed in various types of subpopulations in communities affected by disasters, and various aspects of mental health and psychosocial support have been provided.^12^, ^13^, ^14^, ^15^, ^16^, ^17^ In addition, psychiatric disorders such as posttraumatic stress disorder, major depressive disorder, and anxiety disorders worsen or their onset increases after a disaster.^18^, ^19^, ^20^, ^21^, ^22^ These findings have been accumulated through studies conducted during the chronic phase (months after disaster onset), as it is difficult to examine the mental health of disaster victims immediately after a disaster, following the rigorous procedures required for academic papers. However, it is preferable to accumulate knowledge regarding the mental health conditions and support needs of affected people in the acute phase (e.g., within a month) after the disaster onset to improve the measures for disaster response if it is possible to collect information in a way that avoids negative effects or burdens on the affected people and people responding to the situation. In this study, we used the Emergency Medical Information System (EMIS) data from DPAT activities during the 2024 Noto Peninsula earthquake (NPE) to investigate people's mental health needs during the acute phase of the postdisaster situation, when advanced DPATs were dispatched. The EMIS is designed to “collect and provide appropriate information” in times of disaster and has been developed based on lessons learned from the 1995 GHAE. It is operated by the MHLW and shares information related to disaster medical care, such as the operational status of medical institutions beyond disaster‐affected prefectures, and consolidates and provides information related to appropriate medical care in disaster areas.^23^, ^24^ In this study, we used information accumulated by the EMIS to obtain an overview of mental health needs in the acute phase, up to 1 month after the 2024 NPE.

## METHODS

### Design

This retrospective study examined the content of medical response requests and response details using the EMIS and chronology data (a chronological record and organization of the situation and activities during a disaster emergency) used by the DPAT during its activities after the 2024 NPE, particularly in the affected area covered by our DPAT team (Suzu City and Noto Town). Permission to use the EMIS and chronology data for a period of ∼1 month (January 7 to February 7, 2024) was obtained from the DPAT secretariat of the MHLW.

### Survey items

From the EMIS and chronology data, the following information was extracted for the supportees subjected to the following analyses: age, age groups (teens–20s, 30s–40s, 50s–60s, and >70 years), sex (male, female), consultation stage of DPAT response (first‐time, follow‐up), content of response (medical consultation, telephonic consultation, consultation and medication prescription, arrangement for hospitalization, arranging welfare facilities, arranging an evacuation area, and consultation with support workers), psychiatric treatment history (under treatment, past treatment, no treatment history, and unidentified), and diagnostic classification (International Statistical Classification of Diseases and Related Health Problems 10th Revision [ICD‐10]: F0–F9, other problem, and unidentified). In addition, the rule of one count per consultation was used to compile data. Therefore, in cases where the content of the consultation overlapped in multiple areas, the main consultation (the one that improved the supportee's state the most) was classified as the main consultation. The diagnosis code and corresponding mental and behavioral disorders were as follows: F0, organic, including symptomatic, mental disorders (mainly includes various forms of major and mild neurocognitive disorders); F1, mental and behavioral disorders due to psychoactive substance use; F2, schizophrenia, schizotypal, and delusional disorders; F3, mood (affective) disorders; F4, neurotic, stress‐related, and somatoform disorders; F5, behavioral syndromes associated with physiological and physical disturbances; F6, adult personality and behavior disorders; F7, mental retardation; F8, psychological development disorders; and F9, behavioral and emotional disorders with onset usually occurring in childhood and adolescence. These were not preset classifications but were later classified based on the EMIS and chronology data. In cases where multiple ICD‐10 diagnoses were listed, the diagnosis that most significantly affected the state of the supportee was treated as the primary diagnosis. The EMIS and chronology data used in this study did not include any personal information of the supportees or supporters. All data were anonymized and kept confidential. In addition, aftershock data (January 1–February 8, 2024) at the seismic station in Suzu City were obtained from the strong‐motion seismograph Network of the National Research Institute for Earth Science and Disaster Resilience.^25^

This study was approved by the Ethics Committee of the International Research Institute of Disaster Science, Tohoku University (number: 2023‐055) and was conducted in accordance with the Declaration of Helsinki. Because this was a retrospective medical record survey, the requirement for informed consent was waived; however, information about the study was made available to allow patients the opportunity to freely opt out.

### Statistical analysis

The supportees' variables were compared between the 2 weeks immediately after the disaster (Phase 1: January 7–21) and the following 2 weeks (Phase 2: January 22–February 7). For age, the Mann–Whitney *U* test was used, excluding cases of unknown age. For other categories, the *χ* ^2^ and Fisher's exact tests were used to analyze the frequency of subcategory items. For categories identified as significantly different following a *χ* ^2^ or Fisher's exact test (the significance level was set at P = 0.05), we applied the Benjamini–Hochberg procedure (the significance level was set at *Q* = 0.05) to control for multiple comparisons. Items for which differences were found were further examined for differences in the subitems. IBM SPSS (version 29.0.1) was used for data analysis.

## RESULTS

In total, 186 consultations were conducted during the survey period: 102 medical consultation cases, four telephonic consultation cases, 28 consultation and medication prescription cases, four arrangement for hospitalization cases, three cases of arranging welfare facilities, one case of arranging an evacuation area, seven cases of consultation with support workers, and 37 cases of information sharing with support workers. In addition, the rule of one count per consultation was used to compile data. Therefore, in cases where the content of the consultation overlapped in multiple areas, the main consultation (the one that improved the supportee's state the most) was classified as the main consultation. Consequently, 149 cases were included in the analysis, excluding 37 cases of information sharing with support workers.

As shown in Figure 1, the survey period of this study was divided into Phase 1 (January 7–January 21, 2024) and Phase 2 (January 22–February 7, 2024) for comparison. Statistically significant differences were found for “age and age groups,” “consultation stage,” “psychiatric treatment history,” and “diagnostic classification” (Table 1). However, no differences were found for “sex” and “content of responses.”

**Table 1:** Demographic data and consultation characteristics for each phase.

	Phase 1 (*n* = 112)		Phase 2 (*n* = 37)		*p* value	*Q* value
	Median		Median			
Age (years)^a^	65.00		77.50		0.006^b^^,^ *	0.021
	Frequency	Proportion (%)	Frequency	Proportion (%)		
*Age group* c						
10–29 years	3	2.7	1	2.7	0.034^d^^,^ *	0.048
30–49 years	14	12.5	2	5.4		
50–69 years	31	27.7	6	16.2		
70–99 years	31	27.6	21	56.8		
Unidentified	33	29.5	7	18.9		
*Sex*						
Male	39	34.8	15	40.5	n.s.^d^	‐
Female	68	60.7	19	51.4		
Unidentified	5	4.5	3	8.1		
*Consultation stage*						
First‐time consultation	94	83.9	20	54.1	<0.001^e^^,^ *	<0.007
Follow‐up consultation	18	16.1	17	45.9		
*Content of responses*						
Medical consultation	76	67.9	26	70.3	n.s.^d^	‐
Telephonic consultation	4	3.6	0	0		
Consultation and medication prescription	20	17.9	8	21.6		
Arrangement for hospitalization	4	3.6	0	0		
Arranging welfare facility	3	2.7	0	0		
Arranging an evacuation area	1	0.9	0	0		
Consultation with support worker	4	3.6	3	8.1		
*Psychiatric treatment history*						
Under treatment	46	41.1	5	13.5	0.010^d^^,^ *	0.023
Past treatment	3	2.7	1	2.7		
No treatment history	30	26.8	17	45.9		
Unidentified	33	29.5	14	37.8		
*Diagnostic classification*						
F0	9	8.0	12	32.4	0.010^d^^,^ *	0.018
F1	3	2.7	1	2.7		
F2	18	16.1	4	10.8		
F3	16	14.3	2	5.4		
F4	11	9.8	8	21.6		
F5	0	0	0	0		
F6	2	1.8	0	0		
F7	5	4.5	0	0		
F8	4	3.6	0	0		
F9	0	0	0	0		
Other problem	30	26.8	5	13.5		
Unidentified	14	12.5	5	13.5		

^a^ Participants of unidentified age were excluded from the test (Phase1: 42 participants, Phase2: 7 participants).

^b^ Mann–Whitney *U* test was used.

^c^ Participants of unidentified age were excluded from the test (Phase1: 33 participants, Phase2: 7 participants).

^d^ Fisher's exact test was used.

^e^ *χ* ^2^ test was used.

* *p* values that are significant after Benjamini–Hochberg adjustment.

### Age and age groups

The age was higher in Phase 2 than in Phase 1 (medians: 65 vs. 77.5, P = 0.006, *Q* = 0.021; Table 1). The frequency of the age groups was significantly different between the two phases (P = 0.034, *Q* = 0.048; Table 1;), with the 70–99 age group increasing from 27.6% in Phase 1 to 56.8% in Phase 2 in frequency (P = 0.001; Figure S1).

### Consultation stage

The frequency of first‐time consultations was higher in Phase 1 and follow‐up consultations were more frequent in Phase 2 (P < 0.001, *Q* < 0.007; Table 1 and Figure S2). Of the 112 consultations in Phase 1, 94 were completed as first‐time consultations and 18 were follow‐up consultations (five were follow‐up consultations three or more times). Thirty‐seven consultations in Phase 2 were completed as first‐time consultations and 17 were follow‐up consultations (seven were follow‐up consultations three or more times). All 17 cases were first‐time consultations in Phase 1 and all consultations in Phase 2 were completed as first‐time consultations. Details of the follow‐up consultations in Phases 1 and 2 are shown in Table 2.

**Table 2:** Details of follow‐up consultations for each phase.

		Phase 1	Phase 2
Number of follow‐up consultations		18	17
Detailed description of consultation			
Content of responses	Medical consultation	11	15
	Teleconsultation	0	0
	Consultation and medication prescription	6	2
	Arrangement for hospitalization	1	0
	Arranging welfare facility	0	0
	Arranging an evacuation area	0	0
	Consultation with support worker	0	0
Diagnostic classification	F0	1	6
	F1	1	0
	F2	6	4
	F3	2	0
	F4	1	2
	F5	0	0
	F6	1	0
	F7	0	0
	F8	2	0
	F9	0	0
	Other problem^a^	2	2
	Unidentified^b^	2	3

*Note*: Details of the cases were classified as follow‐up consultations in the consultation stage (Phase 1 = 18, Phase 2 = 17). Summary of the responses and diagnostic classifications.

^a^ Details of other problem are as follows (Phase1: suicide ideation = 2; Phase2: physical disease = 1, family conflict = 1).

^b^ Unable to classify due to insufficient information.

### Psychiatric treatment history

The frequency of patients with a history of psychiatric treatment differed between the two phases (P = 0.01, *Q* = 0.023; Table 1). The percentage of patients undergoing psychiatric treatment decreased from 41.1% in Phase 1 to 13.5% in Phase 2 (P = 0.0002; Figure S3), whereas the percentage of those with no treatment history increased from 26.8% in Phase 1 to 45.9% in Phase 2 (P = 0.03; Figure S3). There was no significant difference in the frequency of past treatment (2.7% in Phase 1, 2.7% in Phase 2) or unidentified (29.5% in Phase 1, 37.8% in Phase 2).

### Diagnostic classification

The diagnostic classification of the participants' psychiatric disorders differed between the two phases (P = 0.01, *Q* = 0.018; Table 1). F0 disorders increased from 8% in Phase 1 to 32.4% in Phase 2 (P = 0.001; Figure S4). F4 disorders increased from 9.8% in Phase 1 to 21.6% in Phase 2, with a marginally significant P value (P = 0.062; Figure S4). Details of “other problem and unidentified” in Phase 1 and Phase 2 are shown in Table 3.

**Table 3:** Details of consultations classified as “other problem” and “unidentified” in the diagnostic classification.

	Phase 1	Phase 2
Number of cases classified as “other problems”	30	5
Detailed description		
Physical disease	9	1
Suicide ideation	4	1
Insomnia	4	1
Relationships within evacuation shelter	4	0
Fatigue	2	1
Workload	2	0
Concerns about their children	2	0
Concerns about impossibility of prenatal checkup	1	0
Family conflict	1	1
Concerns about developing trauma symptoms	1	0
Number of cases classified as “unidentified”^a^	14	5

*Note*: Details of the cases classified as those outside F0–F9 in the diagnostic classification. Consultations and number of cases in each phase.

^a^ Unable to classify due to insufficient information.

## DISCUSSION

This study was the first trial to utilize information regarding DPAT activity in response to the 2024 NPE collected in the EMIS and chronology data to obtain an overview of the mental health needs of the victims during the acute phase of postdisaster settings. We found that during the first month after the disaster (1) new consultations started mostly in the first 2 weeks and dropped thereafter; (2) consultations were initially dominated by victims with existing psychiatric disorders, but over time the number of consultations by those without past psychiatric treatment increased; (3) dementia‐related problems (F0 in ICD‐10) increased and surfaced over time; and (4) direct stress reactions (F4 in ICD‐10) due to the disaster also increased over time. These results demonstrate the usability of EMIS data not only for real‐time understanding of mental health needs, but also for analyzing the accumulated data afterward to extract information useful for reviewing the content, effectiveness, or problems of previous support activities or preparing for the next disaster.

During the 72 h immediately following a disaster, which is considered the time limit for determining life and death, the first and foremost priority should be to save lives. In addition, even days and weeks after a disaster, extreme conditions may persist. Therefore, conducting a research survey in the midst of real chaos can not only cause an ethical issue considering the potential invasion and burden on the victims, but can also impede relief activities and technical difficulties in conducting the survey while ensuring the safety of the investigators. For these reasons, surveys or studies to assess the mental health conditions of victims have been conducted months after a disaster.^26^, ^27^, ^28^, ^29^, ^30^ Although it has been shown that psychiatric disorders (such as posttraumatic stress disorder, major depressive disorder, and anxiety disorders) worsen or that the onset of these disorders increases after a disaster, little is known about the transition of mental health status from immediately after a disaster to the acute and subacute phases.

As mentioned above, Japan is one of the world's most disaster‐prone countries, therefore a DPAT system has been established to support psychiatric and mental health activities in affected areas. Outside Japan, mental health care is included in the US National Disaster Medical System,^31^, ^32^, ^33^ which was proposed by President Reagan in 1980, the year after the St. Helen's Volcano explosion, and established in 1984; however, Rodriguez et al. suggested in 2008 that postdisaster mental health services utilization of the victims had been extremely limited.^34^ These situations may explain why research reports on the mental health condition of disaster victims within a month of disaster onset have rarely been reported in countries other than Japan.

Recently, two studies used the Disaster Mental Health Information Support System, a database of consultation records of victims supported by the DPAT during volcanic eruptions, mudslides, floods, and earthquakes from 2013 (when DPAT was launched) to 2016. One study examined the number of consultations with DPAT and the duration of their activities and reported that the number of consultations surged from 0 to 2 days and reached its peak within approximately a week in all disaster conditions.^6^ The latter study investigated the relationship between the type of disaster and psychiatric symptoms, and reported anxiety, sleep problems, mood and affect, and physical symptoms during the acute phase of every disaster.^10^ After publishing these two reports on DPAT activities during the week immediately after the onset of disasters, EMIS replaced the Disaster Mental Health Information Support System as the major recording system for DPAT activities. Since then, data accumulated in the EMIS have not been used for analyses to obtain an overview of the mental health needs of the affected area after the DPAT activities. The current study on DPAT activities over a 1‐month period during the NPE was valuable because it captured changes in mental health needs over time from the acute to subacute phases.

From the perspective of medical support, the NPE had a distinctive aspect that differed from the 1995 GHAE and the 2016 Kumamoto earthquake. The affected area had a very high aging population, with the older adult ratio in the Suzu City and Okunoto areas being over 50% and 48.9%, respectively,^35^ which might be relevant to the phenomenon that many people were reluctant to evacuate to unfamiliar safe inland places far from their residence, regardless of government advocacy. The road network of the Noto Peninsula, which has a large area and low population density, was destroyed by the earthquake, therefore it took a long time to reach each affected area for support and huge efforts were made to cover all areas. Furthermore, 1 month after the disaster, there was no prospect of restoring the water and sewage systems destroyed by the earthquake. For these reasons, the disaster medical assistance teams continued their activities for an unusually long time after the 2024 NPE.^36^ The comparison of the age distribution between the early and the later periods (Phases 1 and 2, respectively) of this survey showed that the percentage of participants aged ≥70 years was significantly higher in Phase 2. Accordingly, a comparison of diagnostic classifications during the survey period showed that dementia‐related problems increased and surfaced over time, partly because the area had a large older adult population. Older adults who were evacuated from their homes or cars immediately after the disaster were identified through the activities of public health nurses, which may have been reflected in the increased number of consultations in Phase 2.^37^ In disaster‐affected areas, older adults are a representative vulnerable population whose mental health effects are significant,^38^ therefore DPAT activities during this disaster may serve as a model case for future support activities in the affected areas of Japan, which is a super‐aged society. In addition, F4 disorders increased in Phase 2, suggesting that stress‐related problems related to the disaster gradually surfaced as victims continued to live in evacuation shelters and faced an uncertain future.

By contrast, the results of the comparison of first‐time or follow‐up consultations and that of the presence or absence of a psychiatric treatment history showed that the ratio of first‐time consultations was higher in Phase 1 and that the contents of these consultations mainly included patients who had a history of psychiatric treatment or were under treatment. The fact that the largest group of people to be supported by the DPAT immediately after the earthquake were those undergoing treatment for psychiatric disorders is pivotal when considering future preparedness for disasters. It has long been recognized that psychiatric patients are among the most vulnerable during disasters,^39^ and this is especially true for patients admitted to psychiatric hospitals that are weak to disasters and emergencies.^40^, ^41^, ^42^ There were no psychiatric hospitals in the affected areas covered by our DPAT team (Suzu City and Noto Town), and only two general hospitals had outpatient psychiatric departments. The decline in psychiatric outpatient function in this affected area may have been a major factor in the results of this study. Nevertheless, it is considered that in DPAT's support activities, focusing on responding to psychiatric patients immediately after a disaster and minimizing the deterioration of their conditions will greatly contribute to stabilizing the mental health of the people in disaster‐affected areas. Considering that the current DPAT support system limits the involvement of their activities in insured medical care services, in preparation for future disasters, it is highly desirable to establish an environment and legal framework that will enable DPAT to provide insured medical care in the affected areas immediately after a disaster in addition to the currently available mental health support.

However, this study had several limitations. First, there was a certain amount of missing data in many of the survey categories because of the nature of data recording in the midst of a chaotic situation. Likewise, the accuracy of the diagnosis can vary because the clinical evaluations were performed in limited and extreme situations, and diagnoses were made tentatively.

In conclusion, we examined the mental health needs during the acute phase of the 2024 NPE using DPAT activity information accumulated in the EMIS to characterize the mental health needs of the affected communities and indicated that in the early stages of a disaster, most of the DPAT resources were devoted to victims with existing psychiatric disorders. The current study demonstrates the usability of the information accumulated in the EMIS for characterizing mental health needs in communities affected by a disaster, depending on the time frame from the onset of the disaster. By accumulating the knowledge extracted from each disaster, it is possible to establish measures commonly available for various disasters or specific to certain types of disasters. These efforts may be effective in improving community resilience in the affected area and provide basic information for future large‐scale disaster support activities.

## AUTHOR CONTRIBUTION

Yasuto Kunii designed the study. Yasuto Kunii, Yumiko Hamaie, Yusuke Utsumi, and Yasuhisa Fukuo conducted the primary EMIS data search and analyzed the data. Yumiko Hamaie and Mizuki Hino created the figures and tables. Yasuto Kunii and Mizuki Hino wrote the first draft of this manuscript. Hiroaki Tomita played a major role in revising and editing the drafts. Yasuto Kunii, Yumiko Hamaie, Mizuki Hino, Yusuke Utsumi, Yasuhisa Fukuo, and Hiroaki Tomita amended or added text to the draft. All the authors contributed to and approved the final manuscript.

## CONFLICT OF INTEREST STATEMENT

The authors declare no conflicts of interest.

## ETHICS APPROVAL STATEMENT

This study was approved by the Ethics Committee of the International Research Institute of Disaster Science, Tohoku University (No. 2023‐055).

## PATIENT CONSENT STATEMENT

Because this was a retrospective medical recordsurvey, the requirement for informed consent was waived; however, information about the study was made available to allow patients the opportunity to freely opt out.

## CLINICAL TRIAL REGISTRATION

This study is not clinical traial.

## Supporting information

Figure S1.

Figure S2.

Figure S3.

Figure S4.

## Data Availability

Data are available from the corresponding authors upon reasonable request.
