# Supplementary figures and images for: Mental health needs in the acute and subacute phases of the 2024 Noto Peninsula earthquake: Emergency Medical Information System data analysis in Disaster Psychiatric Assistance Team activities
Source: PCN Rep. 2025 Mar 17;4(1):e70085. doi: 10.1002/pcn5.70085 (PMC11913620; doi:10.1002/pcn5.70085)

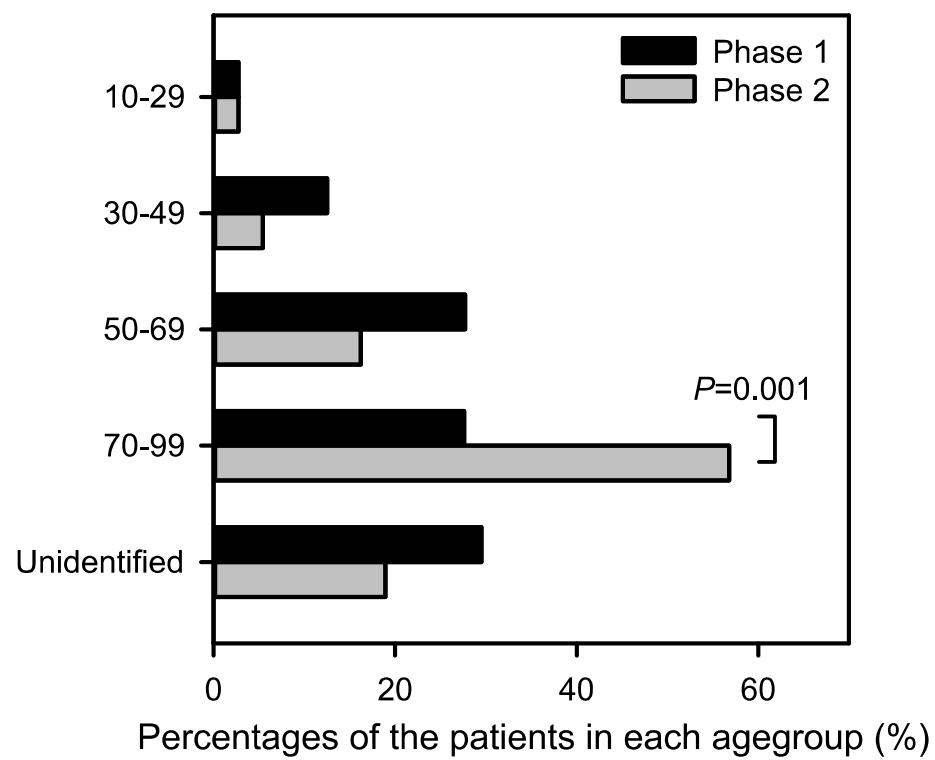

Supplement: Supplementary file 1 — Figure S1. [file PCN5-4-e70085-s002.pdf]

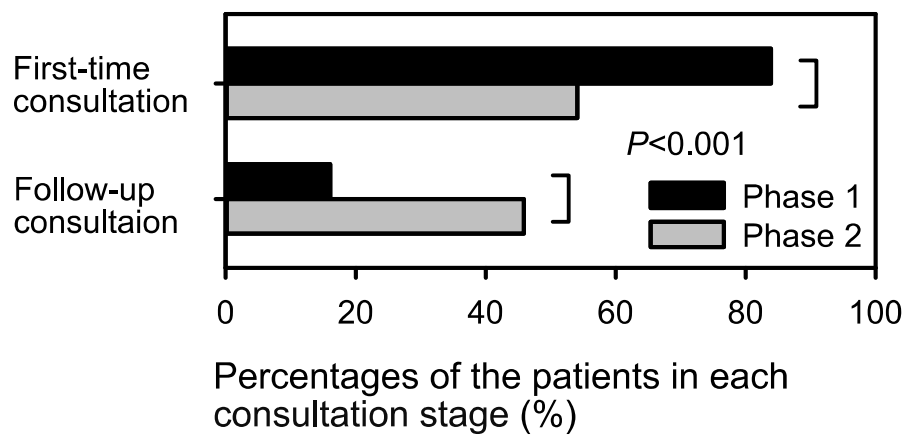

Supplement: Supplementary file 2 — Figure S2. [file PCN5-4-e70085-s001.pdf]

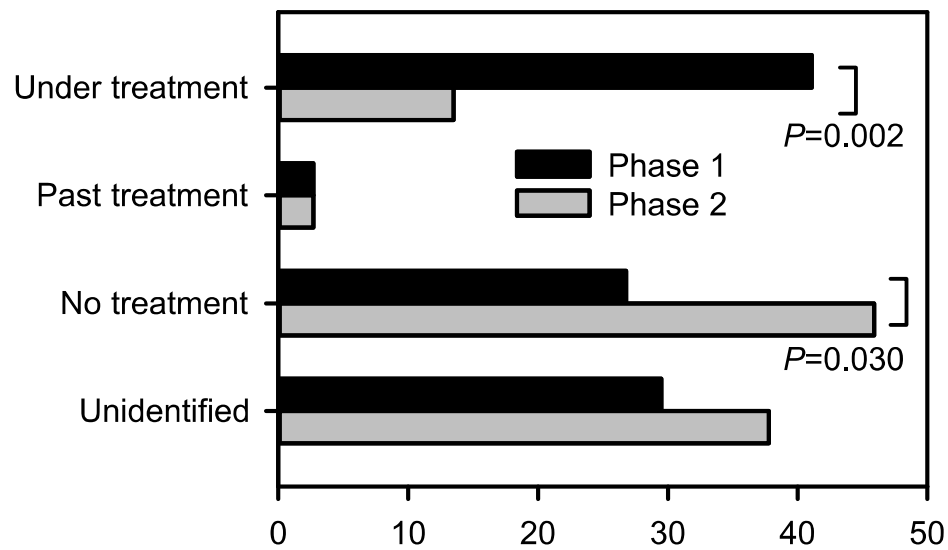

Percentages of the patients in each psychiatric treatment history (%)

Supplement: Supplementary file 3 — Figure S3. [file PCN5-4-e70085-s004.pdf]

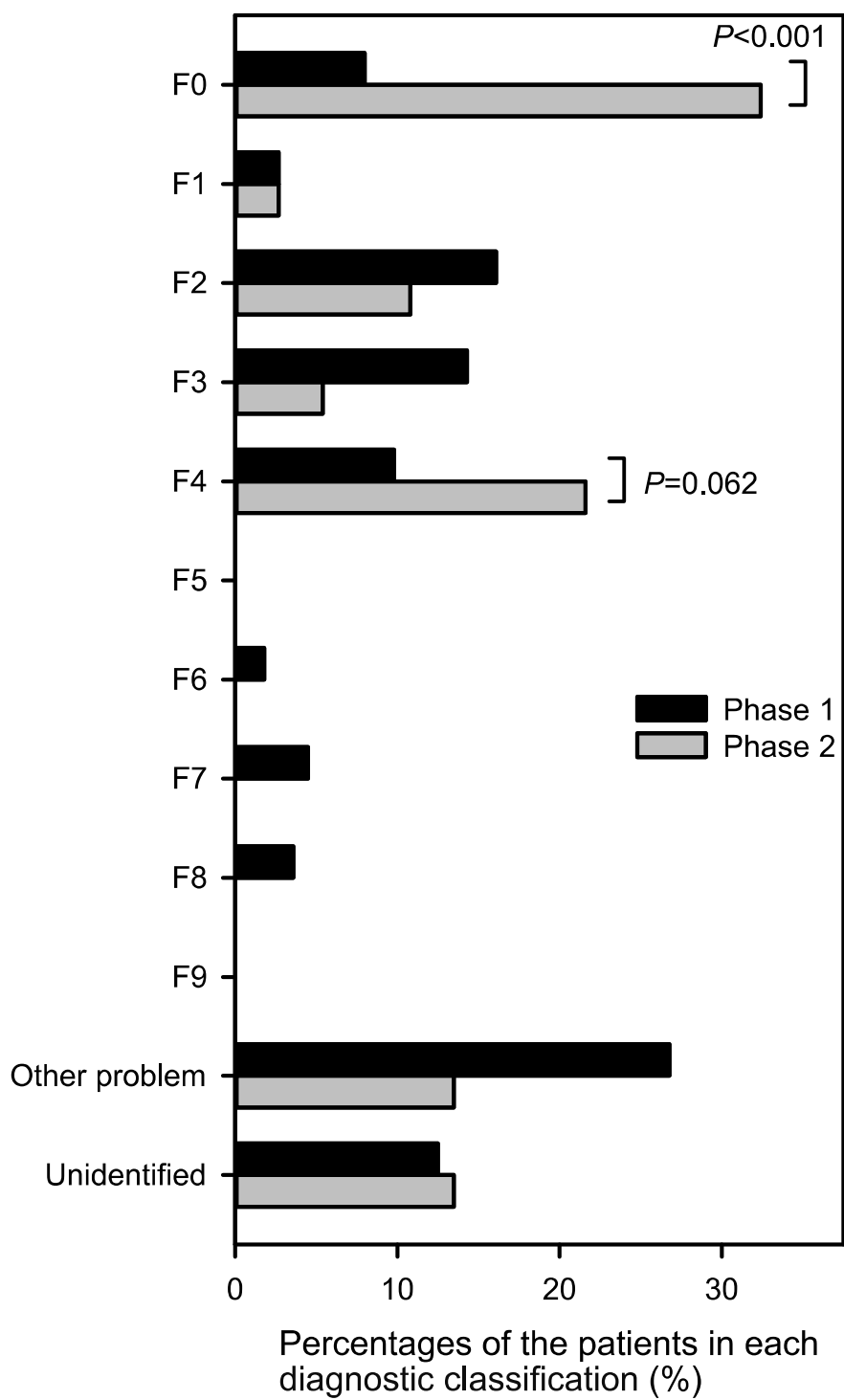

Supplement: Supplementary file 4 — Figure S4. [file PCN5-4-e70085-s003.pdf]
